# Outcome analysis for patients with subarachnoid hemorrhage and vasospasm including endovascular treatment

**DOI:** 10.1186/s42466-023-00283-3

**Published:** 2023-11-02

**Authors:** Sina Burth, Jan Meis, Dorothea Kronsteiner, Helena Heckhausen, Klaus Zweckberger, Meinhard Kieser, Wolfgang Wick, Christian Ulfert, Markus Möhlenbruch, Peter Ringleb, Silvia Schönenberger

**Affiliations:** 1 Department of Neurology, Heidelberg University Hospital, Im Neuenheimer Feld 400, 69120 Heidelberg, Germany; 2 Institute of Medical Biometry, Heidelberg University Hospital, Im Neuenheimer Feld 130.3, 69120 Heidelberg, Germany; 3 Departement of Neurosurgery, Städtisches Klinikum Braunschweig gGmbH, Salzdahlumer Street 90, 38126 Braunschweig, Germany; 4 DKFZ Department of Neurology and Neurooncology Program, National Center for Tumor Diseases, University Hospital Heidelberg, Heidelberg, Germany; 5 German Cancer Consortium (DKTK) and Clinical Cooperation Unit Neurooncology, German Cancer Research Center (DKFZ), Heidelberg, Germany; 6 Department of Neuroradiology, Heidelberg University Hospital, Im Neuenheimer Feld 400, 69120 Heidelberg, Germany

**Keywords:** Subarachnoid hemorrhage, Vasospasm, Delayed cerebral ischemia, Outcome, Endovascular treatment

## Abstract

As a complication of subarachnoid hemorrhage (SAH), vasospasm substantially contributes to its morbidity and mortality. We aimed at analyzing predictors of outcome for these patients including the role of endovascular treatment (ET). Our database was screened for patients with SAH treated in our Neuro-ICU from 2009 to 2019. Clinical parameters including functional outcome (modified Rankin Scale, mRS of 0–2 or 3–6 at discharge and after a median follow-up of 18 months) and details about ET were gathered on 465 patients, 241 (52%) of whom experienced vasospasm. Descriptive analyses were performed to identify explanatory variables for the dichotomized mRS score. A logistic regression model was fitted on 241 patients with vasospasm including age, Hunt and Hess Score, extraventricular drainage (EVD), forced hypertension, ET and delayed cerebral ischemia (DCI). The model found a Hunt and Hess Score of 5 (OR = 0.043, *p* = 0.008), requirement of EVD (OR = 0.161, *p* < 0.001), forced hypertension (OR = 0.242, *p* = 0.001), ET (OR = 0.431, *p* = 0.043) and DCI (OR = 0.229, *p* < 0.001) to be negative predictors of outcome while age was not. Use of intraarterial nimodipine alone (OR = 0.778, *p* = 0.705) or including balloon angioplasty (OR = 0.894, *p* = 0.902) and number of ETs per patient (OR = 0.757, *p* = 0.416) were not significant in a separate model with otherwise identical variables. While DCI is clearly associated with poor outcome, the influence of ET on outcome remains inconclusive. Limited by their retrospective nature and an indication bias, these data encourage a randomized assessment of ET.

**Graphical abstract:**

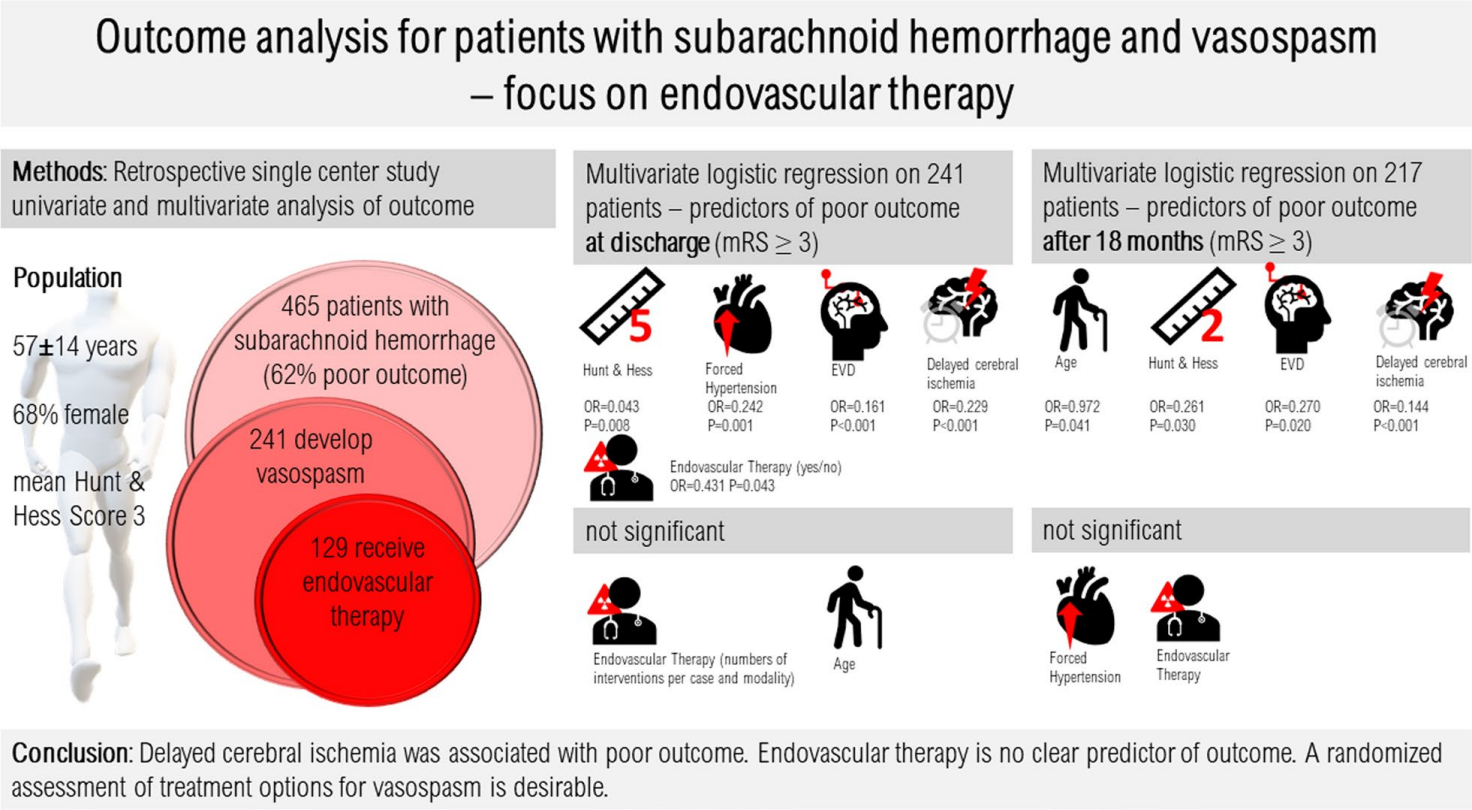

**Supplementary Information:**

The online version contains supplementary material available at 10.1186/s42466-023-00283-3.

## Introduction

Subarachnoid hemorrhage (SAH) is a type of stroke that mainly affects younger people with a mean age of 58 years [30]. Although the outcome has improved within the last decades, SAH still has an in-hospital mortality of 26% [24]. Roughly 25% of the variation in outcome for patients with SAH is explained by known risk factors including older age, worse neurological state on admission, severity of SAH on the initial computed tomography (CT)-scan, a history of hypertension and the size and position of the aneurysm in the posterior circulation [16].

Delayed cerebral ischemia (DCI) due to cerebral vasospasm is a major contributor to morbidity and mortality in patients with SAH and occurs in 20–30% of patients [10]. It is commonly defined as a new neurologic deficit or a decline of 2 points in the Glasgow Coma Scale for at least one hour that cannot be attributed to other causes [36]. Recently, DCI has not been purely attributed to vasospasm but microthrombosis, microcirculatory dysfunction, neuroinflammation, cortical spreading depolarization, and processes triggered by the initial brain injury have been discussed [21, 38]. Known risk factors for the development of vasospasm are the severity of bleeding, younger age (< 50 years), the localization of the aneurysm, hydrocephalus, female sex, hyperglycemia, preexisting hypertension and smoking [5, 7, 9, 20, 22].

Generally, patients with SAH are treated within an intensive care setting and the development of vasospasm, usually peaking around 6–8 days after the onset of SAH [37], is monitored by daily transcranial Doppler-ultrasonography (TCD) and measurement of velocity changes and confirmed by CT angiography (CTA) plus perfusion imaging or digital subtraction angiography (DSA). Standard of care to prevent vasospasm is the administration of oral nimodipine 60 mg every four hours [14, 23, 32]. If vasospasm occurs, guidelines recommend maintenance of euvolemia [23, 32] and potentially escalation to hemodynamic augmentation with vasopressors (after the aneurysm has been clipped or coiled) if necessary [14]. It is unclear if the subsequent use of balloon angioplasty and intra-arterial administration of vasodilators benefits the clinical outcome after the conservative treatments have failed [8] and only the guidelines of the American Heart Association/American Stroke Association recommend ET in cases of severe vasospasm [14], while others do not [23, 32].

In view of the inconclusive findings in literature, we aimed at analyzing predictors of outcome for patients with SAH focusing on the subgroup that develops vasospasm and subsequently receives ET.

## Methods

Adult patients with SAH due to an intracranial aneurysm treated in our neurointensive care unit from 2009 to 2019 were included into this retrospective, single center study and rendered anonymous for further analysis. 552 patients were identified from the administrative database using the corresponding code of the International Statistical Classification of Diseases and Related Health Problems (ICD-10). 87 patients had SAH due to other causes than cerebral aneurysm and were excluded. Extensive data was collected on the remaining 465 patients of which 241 comprised a subgroup that developed vasospasm. The analysis was approved by the local ethics committee (No. S-491/2019).

### Management of patients

All patients were initially admitted to our neurointensive care unit and treated according to our standard of practice (SOP) for aneurysmatic SAH. After initial evaluation with CT and CTA, a DSA was done within 24 h of admission and the treatment modality of the ruptured aneurysm (clipping/coiling) was decided within a multidisciplinary team consisting of a neurologist, a neuroradiologist and a neurosurgeon. Until the aneurysm was treated, systolic blood pressure was kept at < 140 mmHg. All patients received oral Nimodipine 60 mg every four hours and were managed to maintain euvolemia. If necessary, norepinephrine was used as a vasopressor. In patients with hydrocephalus, an EVD was placed and often complemented by a lumbar drain after the aneurysm had been treated. Daily transcranial Doppler-screenings were performed (Dopplerbox X, DWL) or a ptiO2-monitoring (tissue oxygen pressure) was placed. The mean arterial pressure (MAP) was aimed at > 90 mmHg if there was indication of vasospasm. Patients got a multimodal CT if they had 1) a new neurologic deficit not responsive to a raise of the MAP to > 90 mmHg; 2) a mean flow velocity that was increased more than 50 cm/s compared to the contralateral side on TCD [13] or a drop in ptiO2; 3) a mean flow velocity of > 200 cm/s or a Lindegaard ratio of > 6 (indicating severe vasospasm [3, 29]) on TCD. Endovascular treatment was performed if CTA confirmed vasospasm or CT perfusion showed a hypoperfusion in a vessel territory. If CTA and CT perfusion did not show vasospasm but suspicions remained high from a clinical point of view, diagnostic angiography was performed with a possibility to intervene if vasospasm was confirmed there. Endovascular treatment consisted of intraarterial Nimodipine at a dose of 1-3 mg for peripheral vasospasm and / or balloon angioplasty if the DSA showed a narrowing of a proximal vessel of > 50% compared to the contralateral side or previous imaging. There was no significant change in the devices that were used for ET during the observation time. Balloon angioplasty remained the standard of treatment (as opposed to the use of a Comaneci device or self-expanding stents) due to a favorable cost–benefit ratio and as superiority has yet to be established (Badger et al., 2020; [34]). Patients whose aneurysm had been coiled were scheduled for a follow-up CT- or MR-angiography after 6 months. If there was complete occlusion, follow-up appointments took place yearly for 2 years and then in individually defined intervals. If there was incomplete occlusion, an invasive angiography was scheduled. After a clipping, we usually performed an angiography during the initial hospital stay.

### Data collection

The following clinical information was gathered from our electronical database: age, sex, premorbid mRS, NIHSS on admission, cardiovascular risk factors, severity and cause of bleeding including Fisher Scale and Hunt and Hess Score, treatment of the aneurysm (clipping, coiling etc.), number and configuration of aneurysm, vital signs, need for hemodynamic augmentation and vasopressors, administration of nimodipine, presence of hydrocephalus, need for EVD or lumbar drain, seizures, duration of stay, outcome at discharge, outcome at last known follow-up and cause of death. As outcome measure, the mRS was derived both from the discharge letter and from the information in the letter of the last in- or outpatient follow-up at our institution. Poor functional outcome was defined as mRS 3–6, good functional outcome was defined as mRS 0–2. The available written reports were screened for the presence of vasospasm on TCD and/or on CTA/CT-perfusion and a subgroup of 241 patients was formed that had documented vasospasm in any modality for further analysis. Details about endovascular treatment such as the absolute number of interventions per case, the rate of balloon angioplasty, the application of intraarterial nimodipine and complications were gathered. It was noted if there were signs of ischemic stroke on any CT scan after the initial SAH.

### Statistical analysis

All statistical analyses were done using R (version 4.1.3[28]). Data was summarized by descriptive statistics, for continuous variables using mean and standard deviation or median in interquartile range, for categorical variables using absolute and relative frequencies.

Explorative analyses for functional outcome (as defined above) were done using chi-squared tests for categorical variables and Welch’s two-sample t-tests for ordinal and continuous variables. Variables that were statistically significant (p < 0.05) in the explorative analyses were selected for a logistic regression model. To account for the limited cohort size, variables that are relevant for the clinical management of patients in the acute setting (such as placement of EVD, forced hypertension, ET) were prioritized over other clinical variables (such as preexisting hypertension or placement of a ventriculoperitoneal shunt). If variables had a strong intercorrelation or represented similar properties (i.e. Hunt and Hess Score and Fisher Scale; NIHSS and mRS; use of vasopressors and forced hypertension), only one variable was included in the multivariate model.

Finally, the logistic regression model was fitted for the dependent variable mRS score (dichotomized to 0–2 vs. 3–6) using the independent variables (covariates) age, Hunt and Hess Score, placement of EVD, endovascular treatment, forced hypertension, and presence of delayed cerebral ischemia. The odds ratio (OR) with corresponding 95% confidence interval (CI) and the two-sided p-value are presented. Since this is an explorative data analysis, all p-values are of descriptive nature.

## Results

### Patient characteristics for the full cohort (with or without vasospasm)

A total of 465 patients were identified that fulfilled the inclusion criteria (compare Sect. “Methods”). Patients had a mean age of 57 ± 14 years and were mostly female (68%). Most aneurysms originated from the anterior communicating artery (41%) or the posterior communicating artery (14%); 80% of aneurysms were coiled and 12% were clipped within 24 h of admission (others received other ETs or went on to receive palliative care). The median Hunt and Hess Score was 3 (20%). 38% of patients had a favorable outcome of mRS ≤ 2 at discharge. After a median of 18 months, it was 47% of patients (out of 409 cases with available follow-up data). In-hospital mortality was 17%.

### Patient characteristics for the subgroup with vasospasm

Of the initial 465 patients, 40% developed vasospasm according to TCD, 28% and 31% had vasospasm in CTA and CT-perfusion yielding 241 patients (52%) that had documented vasospasm in any modality. The summary results of this cohort are displayed in Table 1 separated by good (mRS 0–2) and poor outcome (mRS 3–6) including the descriptive p-values of the respective t-test or chi-squared-test (a complete table with descriptive statistics of all covariates is available in the Additional file 1: Table S1). 129 patients (54%) received one or up to seven ETs after a median of 8 days. Patients that received early endovascular therapy were more likely to need multiple treatments (Spearman’s rank correlation rho -0.23, p = 0.009). But the time of first detection of vasospasm in TCD, CTA or CTP did not correlate with an aggressive endovascular therapy (i.e. the number of ETs per patient). In total, 60% of patients had delayed cerebral ischemia present on any CT after the initial treatment of the aneurysm until discharge. In the subgroup that received no ET, the rate was 46% whereas it was 73% for patients after ET. 29% of patients had a favorable outcome of mRS ≤ 2 at discharge. After a median of 18 months, it was 46% of patients (out of 217 cases with available follow-up data). Follow-up was done in our outpatient clinic in 45% of cases, 15% of patients had another hospital stay in our department, in 10% of cases follow-up data was derived from the discharge letter of a rehabilitation clinic, 13% of patients had no follow-up data available and date of death was noted in 17% of patients. This patient cohort was additionally stratified based on whether they received ET to examine the homogeneity of these two groups. Detailed results are shown in Table 2. Notably, age and sex were similar in the two groups, but patients who went on to receive ET had higher Hunt and Hess Scores and Fisher Grades and were more frequently clipped, needed an EVD or had intraparenchymal bleeding.

**Table 1:** Descriptive statistics stratified by outcome (mRS 0–2 and 3–6 at discharge) on 241 patients

mRS	3–6	0–2	Total	*p*
	(*N* = 170)	(*N* = 71)	(*N* = 241)	
Age				
* N*	170	71	241	0.002^tt2^
Mean ± SD	57 ± 13	51 ± 13	55 ± 13	
Median (Q1, Q3)	57 (49, 65)	53 (44, 58)	55 (47, 63)	
Min–max	16 – 86	17 – 88	16 – 88	
Sex				
Male	45 (26%)	25 (35%)	70 (29%)	0.173^chi2^
Premorbid mRS				
N	170	71	241	0.004^tt2^
0	140 (82%)	66 (93%)	206 (85%)	
1	17 (10%)	4 (6%)	21 (9%)	
2	11 (6%)	1 (1%)	12 (5%)	
3	2 (1%)	0 (0%)	2 (1%)	
Hunt and Hess score				
* N*	170	71	241	< 0.001^tt2^
Mean ± SD	3.4 ± 1.3	2 ± 1.1	3 ± 1.4	
Median (Q1, Q3)	3 (3, 5)	2 (1, 3)	3 (2, 4)	
Min–max	1 – 5	1 – 5	1 – 5	
1	16 (9%)	32 (45%)	48 (20%)	
2	22 (13%)	18 (25%)	40 (17%)	
3	48 (28%)	12 (17%)	60 (25%)	
4	39 (23%)	8 (11%)	47 (20%)	
5	45 (26%)	1 (1%)	46 (19%)	
Aneurysm treatment				
Coiling	127 (75%)	61 (86%)	188 (78%)	0.047^chi2^
Flow diverter	5 (3%)	3 (4%)	8 (3%)	
Web device	3 (2%)	3 (4%)	6 (2%)	
Clipping	32 (19%)	3 (4%)	35 (15%)	
Coiling + Clipping	3 (2%)	1 (1%)	4 (2%)	
Duration of stay Neuro-ICU [days]				
* N*	170	71	241	< 0.001^tt2^
Median (Q1, Q3)	22 (15, 29)	7 (2, 14)	18 (10, 27)	
Min–max	1 – 65	0 – 37	0 – 65	
Forced hypertension				
No	27 (16%)	39 (55%)	66 (27%)	< 0.001^chi2^
Yes	143 (84%)	32 (45%)	175 (73%)	
Endovascular therapy				
No	59 (35%)	53 (75%)	112 (46%)	< 0.001^chi2^
Yes	111 (65%)	18 (25%)	129 (54%)	
Number of ETs per case				
* N*	170	71	241	< 0.001^tt2^
Mean ± SD	1.1 ± 1.2	0.42 ± 0.89	0.93 ± 1.2	
Min–max	0 – 7	0 – 4	0 – 7	
Treatment during ET				
Nimodipine only	57 (34%)	11 (15%)	68 (28%)	
PTA only	5 (3%)	0 (0%)	5 (2%)	
Nimodipine + PTA	44 (26%)	7 (10%)	51 (21%)	
EVD				
No	11 (6%)	40 (56%)	51 (21%)	< 0.001^chi2^
Yes	159 (94%)	31 (44%)	190 (79%)	
Delayed cerebral ischemia				
No	43 (25%)	53 (75%)	96 (40%)	< 0.001^chi2^
Yes	127 (75%)	18 (25%)	145 (60%)	
mRS at final follow-up				
* N*	154	63	217	< 0.001^tt2^
Missing	16 (9%)	8 (11%)	24 (10%)	
Median (Q1, Q3)	4 (2, 6)	0 (0, 2)	3 (1, 5)	
Duration of follow-up [months] (including in-hospital mortality)				
* N*	153	63	216	0.003^tt2^
Missing	17 (10%)	8 (11%)	25 (10%)	
Mean ± SD	19 ± 28	34 ± 33	24 ± 30	
Median (Q1, Q3)	6 (1, 25)	24 (7, 50)	9 (2, 34)	

*ET* endovascular therapy; *EVD* extraventricular drainage; *LD* lumbar drainage; *mRS* modified Rankin Scale; *N* number of patients; *PTA* percutaneous transluminal angioplasty; *Q1/Q3* first/third quartile; *SD* standard deviation

^chi2^Chi-squared test

^tt2^Welch's two-sample t-test

**Table 2:** Descriptive statistics stratified by endovascular therapy on 241 patients

	No ET	ET	Total	*p*
	(*N* = 112)	(*N* = 129)	(*N* = 241)	
Age				
Mean ± SD	54 ± 13	57 ± 13	55 ± 13	0.121^tt2^
Sex				
Male	39 (35%)	31 (24%)	70 (29%)	0.066^chi2^
Premorbid mRS				
*N*	112	129	241	0.393^tt2^
Mean ± SD	0.18 ± 0.52	0.24 ± 0.6	0.21 ± 0.56	
Median (Q1, Q3)	0 (0, 0)	0 (0, 0)	0 (0, 0)	
Hunt and Hess Score				
* N*	112	129	241	< 0.001^tt2^
Mean ± SD	2.6 ± 1.4	3.3 ± 1.3	3 ± 1.4	
Median (Q1, Q3)	3 (1, 4)	3 (2, 5)	3 (2, 4)	
Aneurysm treatment				
Coiling	90 (80%)	98 (76%)	188 (78%)	0.019^chi2^
Clipping	10 (9%)	25 (19%)	35 (15%)	
Coling + Clipping	1 (1%)	3 (2%)	4 (2%)	
Flow diverter	7 (6%)	1 (1%)	8 (3%)	
Web device	4 (4%)	2 (2%)	6 (2%)	
EVD				
No	38 (34%)	13 (10%)	51 (21%)	< 0.001^chi2^
Yes	74 (66%)	116 (90%)	190 (79%)	
Delayed cerebral ischemia				
No	61 (54%)	35 (27%)	96 (40%)	< 0.001^chi2^
Yes	51 (46%)	94 (73%)	145 (60%)	
mRS at discharge				
N	112	129	241	< 0.001^tt2^
Median (Q1, Q3)	3 (1, 5)	5 (4, 5)	5 (2, 5)	
Mean ± SD	3 ± 2.2	4.5 ± 1.5	3.8 ± 2	
mRS at final follow-up				
* N*	96	121	217	< 0.001^tt2^
Missing	16 (14%)	8 (6%)	24 (10%)	
Mean ± SD	2.4 ± 2.3	3.5 ± 2	3 ± 2.2	
Median (Q1, Q3)	2 (0, 5)	4 (2, 5)	3 (1, 5)	

*ET* endovascular therapy; *EVD* extraventricular drainage; *mRS* modified Rankin Scale; *N* number of patients; *Q1/Q3* first/third quartile; *SD* standard deviation

^chi2^Chi-squared test

^tt2^Welch’s two-sample t-test

### Univariate analysis of outcome for patients with vasospasm

The following covariates were associated with poor outcome: age, high premorbid mRS, preexisting hypertension, all scoring systems (mRS, Hunt and Hess Score, NIHSS, Fisher scale), clipping of the aneurysm, intraparenchymal bleeding, length of stay in ICU and on invasive ventilation as well as in the hospital, fever and pneumonia. Invasive therapeutic measures (administration of vasopressors, forced hypertension, balloon angioplasty and intra-arterial administration of vasodilators) were also generally associated with poor outcome. Sex, other cardiovascular risk factors such as smoking, diabetes, high cholesterol, alcoholism, preexisting coronary artery disease and family history did not seem to influence the outcome and neither did the size, localization or side of the aneurysm.

### Multivariate logistic regression of outcome

Table 3 shows the logistic regression model for the dependent variable mRS (dichotomized to 0–2 vs. 3–6) at discharge including 241 patients. Covariates that were observed to be associated with outcome in this model were a Hunt and Hess Score of 5, the need for an EVD, forced hypertension, endovascular therapy and delayed cerebral ischemia. When the number of ETs per case and the modality (intraarterial nimodipine only and nimodipine plus balloon angioplasty) are included in the regression, ET is no longer associated with poor outcome (compare Additional file 1: Table S2).

**Table 3:** Logistic regression model for mRS 0–2 vs. 3–6 at discharge (*N* = 241)

	Odds ratio	Lower CI	Upper CI	*p*-Value
Age	0.977	0.946	1.007	0.131
Hunt and Hess score 2	0.927	0.278	3.167	0.901
Hunt and Hess score 3	0.369	0.106	1.260	0.111
Hunt and Hess score 4	0.291	0.080	1.032	0.056
Hunt and Hess score 5	0.043	0.002	0.318	**0.008**
Endovascular therapy	0.431	0.188	0.973	**0.043**
Forced hypertension	0.242	0.101	0.569	**0.001**
EVD	0.161	0.054	0.449	** < 0.001**
Delayed cerebral ischemia	0.229	0.099	0.511	** < 0.001**

Bold values are significant (*p* < 0.05)

*CI* confidence interval; *EVD* extraventricular drainage; *TCD* transcranial Doppler-ultrasonography

For patients with available follow-up (*N* = 217), results of the regression analysis are displayed in Table 4. This model now shows higher age and delayed cerebral ischemia as predictors for poor outcome, while a high Hunt and Hess Score, endovascular therapy and forced hypertension are no longer statistically significant.

**Table 4:** Logistic regression model for mRS 0–2 vs. 3–6 after a median follow up of 18 months (*N* = 217)

	Odds ratio	Lower CI	Upper CI	*p*-Value
Age	0.972	0.945	0.998	**0.041**
Hunt and Hess score 2	0.261	0.075	0.868	**0.030**
Hunt and Hess score 3	0.440	0.137	1.382	0.160
Hunt and Hess score 4	0.671	0.203	2.196	0.509
Hunt and Hess score 5	0.394	0.110	1.377	0.146
Endovascular therapy	0.624	0.310	1.262	0.187
Forced hypertension	1.168	0.515	2.752	0.715
EVD	0.270	0.086	0.797	**0.020**
Delayed cerebral ischemia	0.144	0.069	0.287	** < 0.001**

Bold values are significant (*p* < 0.05)

*CI* confidence interval; *EVD* extraventricular drainage; *TCD* transcranial Doppler-ultrasonography

## Discussion

This retrospective analysis confirmed that DCI is an independent predictor of poor outcome in a multivariate logistic regression model of patients with SAH and vasospasm and therefore its prevention and treatment is of high importance. Other predictors of poor outcome were older age, a Hunt and Hess Score of five, forced hypertension and placement of EVD while results on ET remained inconclusive.

Besides medical management and prophylaxis with oral nimodipine, treatment of hydrocephalus with EVD, forced hypertension and endovascular treatment of patients that develop vasospasm constitute the key components of caring for patients in our Neuro-ICU.

Currently, only the guidelines of the American Heart Association/American Stroke Association recommend ET in cases of severe vasospasm [14] as does a literature review by Li et al. [20], while European and British guidelines do not [23, 32]. It has been suggested that the pathophysiology of DCI is influenced by multiple factors such as microthrombosis, microcirculatory dysfunction, neuroinflammation etc. [21, 38]. Therefore, DCI might still occur although perfusion of a vessel has been successfully improved by endovascular therapy as is often demonstrated in the immediately postinterventional imaging. There has been proof of safety and technical efficacy of ET [2, 17, 25, 33, 34], even if repeated ETs are performed [2]. In accordance with our results, there are two retrospective cohort studies of one randomized controlled trial by Polin et al. where ET did not benefit the outcome when compared to controls without intervention. Thirty-one patients with vasospasm that received intraarterial papaverine showed no statistical difference in outcome (favorable outcome of mRS ≤ 2 in 45% vs. 58% in the control group)[27]. Similarly, there was no effect on favorable outcome in 38 patients undergoing balloon angioplasty for vasospasm [26]. Another large retrospective study with a control group (*N* = 1057) was performed by Jabbarli et al. [15] from 2005 to 2012. They found that belonging to a cohort with a less aggressive (14.4% vs. 24.4%) and later (8.9 vs. 6 days) ET regimen led to an unfavorable outcome (mRS > 2 at 6 months after discharge for 50.6% vs. 44%) in a multivariate analysis (OR 0.55, p < 0.0001). DCI was also observed more often (29% vs. 20.8%). Our overall rate of ET was even higher at 54%, and we found DCI in 60% of patients and 54% of patients had an unfavorable outcome defined as mRS > 2 (after a median follow-up of 18 months). While the difference in DCI is probably due to different definitions, our outcome measurements roughly correspond to those of Jabbarli et al.’s cohort that was treated more aggressively. This shows that due to the advances in the field of neuroradiology and endovascular procedures over the course of the last years, a more aggressive approach to vasospasm therapy with ET has been established in comprehensive stroke care centers. Concerning our observation time from 2009–2019 it is of note that the median Hunt and Hess Score increased from 2 to 3 while the outcome parameters remained stable.

Sokolowski et al. [31] found in a retrospective, monocentric analysis that for 159 patients treated with ET, only age and smoking were predictors of poor outcome (mRS 3–6, mortality rate 22%) in a multivariate analysis. They examined the need for repeated ET, and as all patients received ET at least once, they did not include it as covariable in the multivariate analysis. Aburto-Murrieta et al. found no difference in outcome when comparing balloon-angioplasty with intraarterial nimodipine in a small cohort of 30 patients [1] and reported good outcome (mRS ≤ 2) for 25% and 45% of patients after 1 year. Similarly, Chalouhi et al. [6] found that 15% of pre-procedure hypodensities were reversed and 60% of patients showed neurological improvement after ET in a retrospective, single center study of 116 patients. As ET was not a predictor of outcome in the univariate analysis, it was not included in the multivariate logistic model. There is one retrospective safety and efficacy study for percutaneous transluminal angioplasty and intra-arterial administration of verapamil by Jun et al. that was conducted from 2003 to 2008 on 546 patients and reported better outcome data in comparison to our study [18]. Vasospasm occurred less often (42% of patients in their cohort vs. 52% of patients in our cohort as found in any imaging modality—TCD, CTA or CT perfusion). ET was performed less often (35% vs. 54%). Outcome was better (61% vs. 46% with a favorable outcome defined as mRS 0–2 at a median follow up of 180 days) and in-hospital mortality was lower (10% vs. 17%). Although outcome was only descriptively analyzed by Jun et al., we assume that the higher rate of patients with a Hunt and Hess Score of 5 in our cohort (19% vs. 8%) is the reason for the different outcome parameters. Khatri et al. reported in a retrospective analysis that after starting ET at their institution and treating 18 of 57 patients that were included in the analysis, severe disability (mRS 5–6 at discharge) and 1-year-mortality showed a non-significant improvement compared to 89 patients that were admitted before ET was offered [19]. Of two available meta-analyses, one did not find a benefit of ET on outcome [35] while the other reported that there was a benefit for ET at least in the subgroup of patients with refractory vasospasm [4]. To sum up, the evidence for a benefit of ET on outcome remains inconclusive and a comparison of our data to other studies is difficult due to the variety of definitions and covariables.

In addition, older age was a predictor for poor outcome in our follow-up analysis which concurs with available data [12]. Furthermore, forced hypertension, which is an established therapeutic measure to treat vasospasm [14], was associated with poor outcome in our analysis and correspondingly, a prematurely halted randomized controlled trial by Gathier et al. reports no benefit and even serious adverse events [11]. This is also reflected in the British and European guidelines that recommend maintenance of euvolemia only [23, 32].

We assume that the worse clinical outcome for patients treated with open neurosurgery (clipping) is mainly due to the fact that the bleeding was more severe in that subgroup (average Hunt and Hess Score and Fisher Scale for patients with clipping was 3.4 and 3.6 vs. 3.0 and 3.4 for the whole collective). We do also prefer clipping over coiling if there is additional intraparenchymal bleeding or a midline shift that has to be treated surgically and obviously clipping will be performed if coiling has failed. 37 of 241 patients with vasospasm (15%) ended up receiving palliative care which might be a possible confounder when measuring outcome. While all of these patients had documented vasospasm in at least one modality, 19 patients did receive ET before they died.

When interpreting these results, the most relevant limitation of our study surely is confounding by indication, meaning the sickest patients are more likely to need and receive invasive therapies. For example, the placement of an EVD was associated with poor outcome but comprises a life-saving measure in case of hydrocephalus and is therefore indispensable. To some extent, adjusting for disease severity by including the Hunt and Hess Score in the multivariate model addresses this issue. But as shown in Table 2, although age and sex were similar, multiple variables of disease severity were worse in the group of patients receiving ET, including the rate of DCI. We refrained from further adjusting for this issue (i.e. by matching the groups according to a Propensity Score) to maintain adequate cohort size. Similarly, we do attribute the higher rate of DCI in patients after ET (73% versus 46%) to confounding by indication. Patients with vasospasm on TCD only did not receive ET unless they developed a neurological deficit or if vasospasm was confirmed by CTA/CTP. Therefore, they are likely to also have lower rates of DCI.

Furthermore, its retrospective nature and the limited size of the cohort are limitations of this study. The chronological order of events and their interrelation were not being considered. However, as we included most major therapeutic components such as the need for an EVD and forced hypertension, our logistic regression model fits the clinical course of patients with SAH well. Still, we had to refrain from including several variables that were significant in the univariate explorative analyses due to the limited cohort size. Furthermore, although patients were treated within pre-set standard of practice protocols, there is always room for individual treatment decisions in contrast to a prospective, controlled study design. Lastly, 10% of patients had no available follow-up data limiting the explanatory power of that logistic regression model.

## Conclusion

In conclusion, this study demonstrates that outcome for patients with SAH is dependent on clinical markers of disease severity and the development of delayed cerebral ischemia. While these data do not qualify impact of ET in that context, they may support a randomized assessment of treatment options, especially as literature is still inconclusive on that matter.

### Supplementary Information

**Additional file 1**: **Table S1**. Descriptive statistics stratified by outcome (mRS 0–2 and 3–6 at discharge) for all variables on 241 patients. **Table S2**. Logistic regression model for mRS 0–2 vs. 3–6 at discharge with number and modality of endovascular therapy (*N* = 241).

## Data Availability

The datasets are available from the corresponding author. Sina Burth, Jan Meis and Silvia Schönenberger had full access to all the data in the study and take responsibility for its integrity and the data analysis.

## Abbreviations

Chi2: Chi-squared test
CI: Confidence interval
CTA: Computed tomography angiography
DCI: Delayed cerebral ischemia
DSA: Digital subtraction angiography
ET: Endovascular therapy
EVD: Extraventricular drainage
GCS: Glasgow Coma Scale
MAP: Mean arterial pressure
mRS: Modified Rankin Scale
PTA: Percutaneous transluminal angioplasty
ptiO2: Tissue oxygen pressure
SOP: Standard of practice
TCD: Transcranial Doppler-ultrasonography
TT2: Welch's two-sample t-test
