# Supplementary material for: Outcome analysis for patients with subarachnoid hemorrhage and vasospasm including endovascular treatment
Source: Neurol Res Pract. 2023 Nov 2;5:57. doi: 10.1186/s42466-023-00283-3 (PMC10621117; doi:10.1186/s42466-023-00283-3)
Supplement: Supplementary file 1 — Additional file 1: Table S1. Descriptive statistics stratified by outcome (mRS 0–2 and 3–6 at discharge) for all variables on 241 patients. Table S2. Logistic regression model for mRS 0–2 vs. 3–6 at discharge with number and modality of endovascular therapy (N = 241). [file 42466_2023_283_MOESM1_ESM.docx]

*Table S1: Descriptive statistics stratified by outcome (mRS 0-2 and 3-6 at discharge) for all variables on 241 patients*

| **mRS** | **3-6** | **0-2** | **total** | **p** |
| --- | --- | --- | --- | --- |
|  | (N=170) | (N=71) | (N=241) |  |
| **age** |  |  |  |  |
| N | 170 | 71 | 241 | 0.002^tt2^ |
| mean ± sd | 57 ± 13 | 51 ± 13 | 55 ± 13 |  |
| median (Q1, Q3) | 57 (49, 65) | 53 (44, 58) | 55 (47, 63) |  |
| min - max | 16 -- 86 | 17 -- 88 | 16 -- 88 |  |
| **sex** |  |  |  |  |
| male | 45 (26%) | 25 (35%) | 70 (29%) | 0.173^chi2^ |
| **premorbid mRS** |  |  |  |  |
| N | 170 | 71 | 241 | 0.004^tt2^ |
| 0 | 140 (82%) | 66 (93%) | 206 (85%) |  |
| 1 | 17 (10%) | 4 (6%) | 21 (9%) |  |
| 2 | 11 (6%) | 1 (1%) | 12 (5%) |  |
| 3 | 2 (1%) | 0 (0%) | 2 (1%) |  |
| **NIHSS at discharge** |  |  |  |  |
| N | 152 | 66 | 218 | <0.001^tt2^ |
| Missing | 18 (11%) | 5 (7%) | 23 (10%) |  |
| mean ± sd | 30 ± 14 | 0.45 ± 1.4 | 21 ± 18 |  |
| median (Q1, Q3) | 38 (21, 42) | 0 (0, 0) | 24 (0, 38) |  |
| min - max | 0 -- 42 | 0 -- 7 | 0 -- 42 |  |
| **Hunt and Hess Score** |  |  |  |  |
| N | 170 | 71 | 241 | <0.001^tt2^ |
| mean ± sd | 3.4 ± 1.3 | 2 ± 1.1 | 3 ± 1.4 |  |
| median (Q1, Q3) | 3 (3, 5) | 2 (1, 3) | 3 (2, 4) |  |
| min - max | 1 -- 5 | 1 -- 5 | 1 -- 5 |  |
| 1 | 16 (9%) | 32 (45%) | 48 (20%) |  |
| 2 | 22 (13%) | 18 (25%) | 40 (17%) |  |
| 3 | 48 (28%) | 12 (17%) | 60 (25%) |  |
| 4 | 39 (23%) | 8 (11%) | 47 (20%) |  |
| 5 | 45 (26%) | 1 (1%) | 46 (19%) |  |
| **Fisher Grade** |  |  |  |  |
| N | 167 | 65 | 232 | <0.001^tt2^ |
| Missing | 3 (2%) | 6 (8%) | 9 (4%) |  |
| mean ± sd | 3.5 ± 0.67 | 3 ± 0.96 | 3.4 ± 0.79 |  |
| median (Q1, Q3) | 4 (3, 4) | 3 (2, 4) | 4 (3, 4) |  |
| min - max | 1 -- 4 | 1 -- 4 | 1 -- 4 |  |
| 0 | 0 (0%) | 0 (0%) | 0 (0%) |  |
| 1 | 1 (1%) | 5 (8%) | 6 (3%) |  |
| 2 | 13 (8%) | 14 (22%) | 27 (12%) |  |
| 3 | 48 (29%) | 21 (32%) | 69 (30%) |  |
| 4 | 105 (63%) | 25 (38%) | 130 (56%) |  |
| **Localisation of aneurysm** |  |  |  |  |
| A. com. anterior | 59 (35%) | 31 (44%) | 90 (37%) | 0.175^chi2^ |
| A. cerebri media | 16 (9%) | 5 (7%) | 21 (9%) |  |
| A. com. posterior | 24 (14%) | 13 (18%) | 37 (15%) |  |
| A. basilaris | 11 (6%) | 6 (8%) | 17 (7%) |  |
| A. cerebelli post. inferior | 9 (5%) | 1 (1%) | 10 (4%) |  |
| A. carotis interna | 19 (11%) | 5 (7%) | 24 (10%) |  |
| A. cerebri ant. | 3 (2%) | 2 (3%) | 5 (2%) |  |
| A. vertebralis | 3 (2%) | 0 (0%) | 3 (1%) |  |
| A. cerebri post. | 4 (2%) | 0 (0%) | 4 (2%) |  |
| A. cerebelli sup. | 0 (0%) | 2 (3%) | 2 (1%) |  |
| Others | 5 (3%) | 3 (4%) | 8 (3%) |  |
| Multiple sites | 17 (10%) | 3 (4%) | 20 (8%) |  |
| **side** |  |  |  |  |
| right | 57 (42%) | 23 (43%) | 80 (42%) | 0.989^chi2^ |
| left | 53 (39%) | 20 (37%) | 73 (38%) |  |
| Posterior circulation | 18 (13%) | 8 (15%) | 26 (14%) |  |
| A. com. ant. | 8 (6%) | 3 (6%) | 11 (6%) |  |
| Missing | 34 | 17 | 51 |  |
| **Size of aneurysm in mm** |  |  |  |  |
| </=4mm | 48 (30%) | 20 (30%) | 68 (30%) | 0.937^chi2^ |
| >4mm | 110 (70%) | 47 (70%) | 157 (70%) |  |
| Missing | 12 | 4 | 16 |  |
| **Aneurysm treatment** |  |  |  |  |
| Coiling | 127 (75%) | 61 (86%) | 188 (78%) | 0.047^chi2^ |
| Flow diverter | 5 (3%) | 3 (4%) | 8 (3%) |  |
| Web device | 3 (2%) | 3 (4%) | 6 (2%) |  |
| Clipping | 32 (19%) | 3 (4%) | 35 (15%) |  |
| Coiling + Clipping | 3 (2%) | 1 (1%) | 4 (2%) |  |
| **Duration of stay Neuro-ICU [days]** |  |  |  |  |
| N | 170 | 71 | 241 | <0.001^tt2^ |
| median (Q1, Q3) | 22 (15, 29) | 7 (2, 14) | 18 (10, 27) |  |
| min - max | 1 -- 65 | 0 -- 37 | 0 -- 65 |  |
| **Duration of stay stroke unit [days]** |  |  |  |  |
| N | 170 | 71 | 241 | <0.001^tt2^ |
| median (Q1, Q3) | 0 (0, 0) | 5 (0, 9) | 0 (0, 3) |  |
| min - max | 0 -- 11 | 0 -- 15 | 0 -- 15 |  |
| **Duration of stay in hospital [days]** |  |  |  |  |
| N | 170 | 71 | 241 | 0.010^tt2^ |
| median (Q1, Q3) | 24 (16, 29) | 18 (14, 25) | 22 (15, 29) |  |
| min - max | 2 -- 67 | 1 -- 56 | 1 -- 67 |  |
| **Invasive ventilation [days]** |  |  |  |  |
| N | 170 | 71 | 241 | <0.001^tt2^ |
| median (Q1, Q3) | 20 (11, 28) | 0 (0, 2) | 14 (2, 26) |  |
| min - max | 0 -- 65 | 0 -- 32 | 0 -- 65 |  |
| **Vasospasm present on TCD** |  |  |  |  |
| no | 49 (29%) | 5 (7%) | 54 (22%) | <0.001^chi2^ |
| yes | 121 (71%) | 66 (93%) | 187 (78%) |  |
| **Vasospasm present on CTA** |  |  |  |  |
| no | 63 (37%) | 49 (69%) | 112 (46%) | <0.001^chi2^ |
| yes | 107 (63%) | 22 (31%) | 129 (54%) |  |
| **Vasospasm present on CT-perfusion** |  |  |  |  |
| no | 47 (28%) | 50 (70%) | 97 (40%) | <0.001^chi2^ |
| yes | 123 (72%) | 21 (30%) | 144 (60%) |  |
| **Forced hypertension** |  |  |  |  |
| no | 27 (16%) | 39 (55%) | 66 (27%) | <0.001^chi2^ |
| yes | 143 (84%) | 32 (45%) | 175 (73%) |  |
| **Endovascular therapy** |  |  |  |  |
| no | 59 (35%) | 53 (75%) | 112 (46%) | <0.001^chi2^ |
| yes | 111 (65%) | 18 (25%) | 129 (54%) |  |
| **Number of ETs per case** |  |  |  |  |
| N | 170 | 71 | 241 | <0.001^tt2^ |
| mean ± sd | 1.1 ± 1.2 | 0.42 ± 0.89 | 0.93 ± 1.2 |  |
| min - max | 0 -- 7 | 0 -- 4 | 0 -- 7 |  |
| **Treatment during ET** |  |  |  |  |
| Nimodipine only | 57 (34%) | 11 (15%) | 68 (28%) |  |
| PTA only | 5 (3%) | 0 (0%) | 5 (2%) |  |
| Nimodipine + PTA | 44 (26%) | 7 (10%) | 51 (21%) |  |
| **Complications of ET** |  |  |  |  |
| no | 160 (94%) | 69 (97%) | 229 (95%) | 0.319^chi2^ |
| yes | 10 (6%) | 2 (3%) | 12 (5%) |  |
| **Orale nimodipine** |  |  |  |  |
| no | 4 (2%) | 1 (1%) | 5 (2%) | 0.639^chi2^ |
| yes | 166 (98%) | 70 (99%) | 236 (98%) |  |
| **EVD** |  |  |  |  |
| no | 11 (6%) | 40 (56%) | 51 (21%) | <0.001^chi2^ |
| yes | 159 (94%) | 31 (44%) | 190 (79%) |  |
| **Duration of EVD [days]** |  |  |  |  |
| N | 168 | 71 | 239 | <0.001^tt2^ |
| Missing | 2 (1%) | 0 (0%) | 2 (1%) |  |
| median (Q1, Q3) | 14 (9, 20) | 0 (0, 6) | 11 (5, 18) |  |
| min - max | 0 -- 47 | 0 -- 23 | 0 -- 47 |  |
| **Lumbar drainage** |  |  |  |  |
| no | 88 (52%) | 44 (62%) | 132 (55%) | 0.147^chi2^ |
| yes | 82 (48%) | 27 (38%) | 109 (45%) |  |
| **Duration of LD [days]** |  |  |  |  |
| N | 167 | 71 | 238 | 0.074^tt2^ |
| Missing | 3 (2%) | 0 (0%) | 3 (1%) |  |
| median (Q1, Q3) | 0 (0, 8) | 0 (0, 5) | 0 (0, 7) |  |
| min - max | 0 -- 25 | 0 -- 25 | 0 -- 25 |  |
| **Delayed cerebral ischemia** |  |  |  |  |
| no | 43 (25%) | 53 (75%) | 96 (40%) | <0.001^chi2^ |
| yes | 127 (75%) | 18 (25%) | 145 (60%) |  |
| **Intraparenchymal bleeding** |  |  |  |  |
| no | 125 (74%) | 65 (92%) | 190 (79%) | 0.002^chi2^ |
| yes | 45 (26%) | 6 (8%) | 51 (21%) |  |
| **Re-Bleed** |  |  |  |  |
| no | 139 (82%) | 66 (93%) | 205 (85%) | 0.026^chi2^ |
| yes | 31 (18%) | 5 (7%) | 36 (15%) |  |
| **seizures** |  |  |  |  |
| no | 146 (86%) | 66 (93%) | 212 (88%) | 0.124^chi2^ |
| yes | 24 (14%) | 5 (7%) | 29 (12%) |  |
| **Systolic BP on admission [mmHg]** |  |  |  |  |
| N | 166 | 70 | 236 | 0.172^tt2^ |
| Missing | 4 (2%) | 1 (1%) | 5 (2%) |  |
| median (Q1, Q3) | 140 (120, 170) | 152 (138, 160) | 145 (130, 168) |  |
| min - max | 60 -- 220 | 100 -- 220 | 60 -- 220 |  |
| **Diastolic BP on admission [mmHg]** |  |  |  |  |
| N | 166 | 70 | 236 | 0.163^tt2^ |
| Missing | 4 (2%) | 1 (1%) | 5 (2%) |  |
| median (Q1, Q3) | 80 (60, 90) | 80 (70, 90) | 80 (60, 90) |  |
| min - max | 20 -- 150 | 50 -- 140 | 20 -- 150 |  |
| **Fever** |  |  |  |  |
| no | 28 (16%) | 27 (38%) | 55 (23%) | <0.001^chi2^ |
| yes | 142 (84%) | 44 (62%) | 186 (77%) |  |
| **Pneumonia** |  |  |  |  |
| no | 74 (44%) | 61 (86%) | 135 (56%) | <0.001^chi2^ |
| yes | 96 (56%) | 10 (14%) | 106 (44%) |  |
| **Alcoholism** |  |  |  |  |
| no | 158 (93%) | 67 (94%) | 225 (93%) | 0.685^chi2^ |
| yes | 12 (7%) | 4 (6%) | 16 (7%) |  |
| **Hypertension** |  |  |  |  |
| no | 85 (50%) | 48 (68%) | 133 (55%) | 0.012^chi2^ |
| yes | 85 (50%) | 23 (32%) | 108 (45%) |  |
| **High cholesterol** |  |  |  |  |
| no | 156 (92%) | 67 (94%) | 223 (93%) | 0.484^chi2^ |
| yes | 14 (8%) | 4 (6%) | 18 (7%) |  |
| **Diabetes** |  |  |  |  |
| no | 159 (94%) | 70 (99%) | 229 (95%) | 0.100^chi2^ |
| yes | 11 (6%) | 1 (1%) | 12 (5%) |  |
| **Smoking** |  |  |  |  |
| no | 130 (76%) | 40 (56%) | 170 (71%) | 0.002^chi2^ |
| yes | 40 (24%) | 31 (44%) | 71 (29%) |  |
| **Family history** |  |  |  |  |
| no | 165 (97%) | 71 (100%) | 236 (98%) | 0.144^chi2^ |
| yes | 5 (3%) | 0 (0%) | 5 (2%) |  |
| **Atrial fibrillation** |  |  |  |  |
| no | 159 (94%) | 69 (97%) | 228 (95%) | 0.252^chi2^ |
| yes | 11 (6%) | 2 (3%) | 13 (5%) |  |
| **Coronary artery disease** |  |  |  |  |
| no | 158 (93%) | 69 (97%) | 227 (94%) | 0.199^chi2^ |
| yes | 12 (7%) | 2 (3%) | 14 (6%) |  |
| **Cause of death** |  |  |  |  |
| cerebral | 39 (85%) | 1 (100%) | 40 (85%) | 0.672^chi2^ |
| extracerebral | 7 (15%) | 0 (0%) | 7 (15%) |  |
| **mRS at final follow-up** |  |  |  |  |
| N | 154 | 63 | 217 | <0.001^tt2^ |
| Missing | 16 (9%) | 8 (11%) | 24 (10%) |  |
| median (Q1, Q3) | 4 (2, 6) | 0 (0, 2) | 3 (1, 5) |  |
| **Duration of follow-up [months]** (including in-hospital mortality) |  |  |  |  |
| N | 153 | 63 | 216 | 0.003^tt2^ |
| Missing | 17 (10%) | 8 (11%) | 25 (10%) |  |
| mean ± sd | 19 ± 28 | 34 ± 33 | 24 ± 30 |  |
| median (Q1, Q3) | 6 (1, 25) | 24 (7, 50) | 9 (2, 34) |  |
| **PtiO2-monitoring** |  |  |  |  |
| no | 124 (73%) | 69 (97%) | 193 (80%) | <0.001^chi2^ |
| yes | 45 (27%) | 2 (3%) | 47 (20%) |  |
| unknown | 1 | 0 | 1 |  |
| **Ventriculoperitoneal shunt placed** |  |  |  |  |
| no | 104 (65%) | 61 (90%) | 165 (72%) | <0.001^chi2^ |
| yes | 56 (35%) | 7 (10%) | 63 (28%) |  |
| unknown | 10 | 3 | 13 |  |
| **Days from event to vasospasm on TCD** |  |  |  |  |
| N | 94 | 59 | 153 | 0.074^tt2^ |
| Missing | 76 (45%) | 12 (17%) | 88 (37%) |  |
| median (Q1, Q3) | 5 (3, 7) | 6 (3, 9) | 5 (3, 8) |  |
| min - max | 0 -- 37 | 1 -- 39 | 0 -- 39 |  |
| **Days from event to vasospasm on CTA** |  |  |  |  |
| N | 106 | 21 | 127 | 0.207^tt2^ |
| Missing | 64 (38%) | 50 (70%) | 114 (47%) |  |
| median (Q1, Q3) | 7 (5, 9) | 8 (6, 11) | 7 (5, 9) |  |
| min - max | 1 -- 38 | 0 -- 39 | 0 -- 39 |  |
| **Days from event to vasospasm on CTP** |  |  |  |  |
| N | 125 | 21 | 146 | 0.383^tt2^ |
| Missing | 45 (26%) | 50 (70%) | 95 (39%) |  |
| median (Q1, Q3) | 7 (5, 10) | 8 (6, 10) | 7 (5, 10) |  |
| min - max | 1 -- 38 | 2 -- 39 | 1 -- 39 |  |
| **Days from event to documented DCI** |  |  |  |  |
| N | 126 | 18 | 144 | 0.434^tt2^ |
| Missing | 44 (26%) | 53 (75%) | 97 (40%) |  |
| median (Q1, Q3) | 10 (6, 13) | 12 (6, 16) | 10 (6, 13) |  |
| min - max | 1 -- 66 | 2 -- 49 | 1 -- 66 |  |
| **Days from event to ET** |  |  |  |  |
| N | 108 | 18 | 126 | 0.436^tt2^ |
| Missing | 62 (36%) | 53 (75%) | 115 (48%) |  |
| median (Q1, Q3) | 8 (6, 10) | 9 (6, 11) | 8 (6, 10) |  |
| min - max | 3 -- 38 | 0 -- 39 | 0 -- 39 |  |
| ^chi2^Chi-squared test; ET: endovascular therapy; EVD: extraventricular drainage; LD: lumbar drainage; mRS: modified Rankin Scale; N: number of patients; PTA: percutaneous transluminal angioplasty; Q1/Q3: first/third quartile; SD: standard deviation; ^tt2^Welch's two-sample t-test  *Table S2: Logistic regression model for mRS 0-2 vs. 3-6 at discharge with number and modality of endovascular therapy (N=241)* | | | | |

|  | **Odds Ratio** | **Lower CI** | **Upper CI** | **p-Value** |
| --- | --- | --- | --- | --- |
| **Age** | 0.976 | 0.945 | 1.006 | 0.127 |
| **Hunt and Hess Score 2** | 0.932 | 0.277 | 3.222 | 0.910 |
| **Hunt and Hess Score 3** | 0.357 | 0.103 | 1.215 | 0.098 |
| **Hunt and Hess Score 4** | 0.295 | 0.082 | 1.039 | 0.057 |
| **Hunt and Hess Score 5** | 0.040 | 0.002 | 0.299 | **0.007** |
| **Forced hypertension** | 0.237 | 0.097 | 0.562 | **0.001** |
| **EVD** | 0.149 | 0.049 | 0.418 | **<0.001** |
| **Delayed cerebral ischemia**  **Number of ETs per case** | 0.231  0.757 | 0.098  0.353 | 0.524  1.392 | **<0.001**  0.416 |
| **Nimodipine only**  **Nimodipine plus PTA** | 0.778  0.894 | 0.213  0.147 | 2.953  5.338 | 0.705  0.902 |

CI: confidence interval; EVD: extraventricular drainage; ET: endovascular therapy; PTA: percutaneous transluminal angioplasty
